# Supplementary material for: Aerobic exercise and action observation priming modulate functional connectivity
Source: PLoS One. 2023 Apr 6;18(4):e0283975. doi: 10.1371/journal.pone.0283975 (PMC10079047; doi:10.1371/journal.pone.0283975)
Supplement: S1 Table — (DOCX) [file pone.0283975.s001.docx]

**S1 Table. Electrode numbers for predefined regions**

| **Predefined Region of Interest** | **Electrode Number** |
| --- | --- |
| Left Primary Motor Cortex | 51, 52, 58, 59, 60, 65, 66 |
| Right Primary Motor Cortex | 155, 164, 182, 183, 184, 195, 196 |
| Supplementary Motor Area | 6, 7, 8, 15, 16, 17, 23, 24, 198, 207 |
| Left Doral Premotor Cortex | 30, 36, 41, 42, 43, 49, 50, 56, 57 |
| Left Parietal Cortex | 76, 77, 85, 86, 87, 88, 89, 96, 97, 98, 99, 100, 106, 107, 108, 109, 110, 118 |

***Electrode numbers correspond to a 256-lead Hydrocel net (Electrogeodesics Inc., Eugene, OR)***
