# Supplementary material for: Aerobic exercise and action observation priming modulate functional connectivity
Source: PLoS One. 2023 Apr 6;18(4):e0283975. doi: 10.1371/journal.pone.0283975 (PMC10079047; doi:10.1371/journal.pone.0283975)
Supplement: S2 Table — (DOCX) [file pone.0283975.s002.docx]

**S2 Table. Alpha (7-12 Hz) Coherence**

|  |  | **Pre** | **Post** | **Post10** | **Post20** | **Post30** |
| --- | --- | --- | --- | --- | --- | --- |
| **A** | **lM1-rM1** | 0.302, 0.099 | 0.246, 0.111 | 0.248, 0.108 | 0.271, 0.110 | 0.266, 0.095 |
|  | **lM1-SMA** | 0.175, 0.081 | 0.213, 0.097 | 0.291, 0.077 | 0.320, 0.101 | 0.300, 0.069 |
|  | **lM1-PMd** | 0.505, 0.134 | 0.506, 0.091 | 0.414, 0.102 | 0.425, 0.088 | 0.411, 0.098 |
|  | **lM1-Pr** | 0.337, 0.142 | 0.270, 0.089 | 0.359, 0.114 | 0.369, 0.110 | 0.364, 0.084 |
|  |  |  |  |  |  |  |
| **AO** | **lM1-rM1** | 0.393, 0.172 | 0.409, 0.179 | 0.306, 0.131 | 0.328, 0.120 | 0.335, 0.140 |
|  | **lM1-SMA** | 0.201, 0.080 | 0.205, 0.079 | 0.335, 0.136 | 0.367, 0.114 | 0.338, 0.140 |
|  | **lM1-PMd** | 0.484, 0.091 | 0.486, 0.071 | 0.437, 0.104 | 0.476, 0.108 | 0.473, 0.125 |
|  | **lM1-Pr** | 0.358, 0.113 | 0.373, 0.126 | 0.389, 0.089 | 0.386, 0.097 | 0.431, 0.105 |

Values presented as mean, standard deviation. A, aerobic exercise priming; AO, action observation priming
