# Supplementary material for: Aerobic exercise and action observation priming modulate functional connectivity
Source: PLoS One. 2023 Apr 6;18(4):e0283975. doi: 10.1371/journal.pone.0283975 (PMC10079047; doi:10.1371/journal.pone.0283975)
Supplement: S3 Table — (DOCX) [file pone.0283975.s003.docx]

**S3 Table. Low Beta (13-19 Hz) Coherence**

|  |  | **Pre** | **Post** | **Post10** | **Post20** | **Post30** |
| --- | --- | --- | --- | --- | --- | --- |
| **A** | **lM1-rM1** | 0.229, 0.115 | 0.212, 0.149 | 0.304, 0.097 | 0.293, 0.085 | 0.273, 0.077 |
|  | **lM1-SMA** | 0.160, 0.105 | 0.221, 0.129 | 0.349, 0.068 | 0.347, 0.091 | 0.323, 0.063 |
|  | **lM1-PMd** | 0.490, 0.133 | 0.492, 0.126 | 0.427, 0.092 | 0.416, 0.090 | 0.376, 0.105 |
|  | **lM1-Pr** | 0.311, 0.145 | 0.236, 0.116 | 0.399, 0.104 | 0.388, 0.083 | 0.372, 0.068 |
|  |  |  |  |  |  |  |
| **AO** | **lM1-rM1** | 0.338, 0.138 | 0.362, 0.163 | 0.314, 0.093 | 0.327, 0.069 | 0.338, 0.098 |
|  | **lM1-SMA** | 0.187, 0.058 | 0.192, 0.080 | 0.345, 0.104 | 0.374, 0.100 | 0.362, 0.141 |
|  | **lM1-PMd** | 0.457, 0.086 | 0.481, 0.066 | 0.426, 0.082 | 0.461, 0.074 | 0.450, 0.131 |
|  | **lM1-Pr** | 0.340, 0.104 | 0.344, 0.146 | 0.382, 0.070 | 0.384, 0.074 | 0.411, 0.078 |

Values presented as mean, standard deviation. A, aerobic exercise priming; AO, action observation priming
