# Supplementary material for: Aerobic exercise and action observation priming modulate functional connectivity
Source: PLoS One. 2023 Apr 6;18(4):e0283975. doi: 10.1371/journal.pone.0283975 (PMC10079047; doi:10.1371/journal.pone.0283975)
Supplement: S4 Table — (DOCX) [file pone.0283975.s004.docx]

**S4 Table. High Beta (20-30 Hz) Coherence**

|  |  | **Pre** | **Post** | **Post10** | **Post20** | **Post30** |
| --- | --- | --- | --- | --- | --- | --- |
| **A** | **lM1-rM1** | 0.167, 0.085 | 0.171, 0.121 | 0.404, 0.117 | 0.349, 0.104 | 0.308, 0.072 |
|  | **lM1-SMA** | 0.144, 0.096 | 0.186, 0.111 | 0.442, 0.120 | 0.412, 0.150 | 0.386, 0.106 |
|  | **lM1-PMd** | 0.437, 0.156 | 0.428, 0.134 | 0.461, 0.099 | 0.420, 0.095 | 0.382, 0.117 |
|  | **lM1-Pr** | 0.282, 0.119 | 0.219, 0.094 | 0.486, 0.092 | 0.451, 0.090 | 0.428, 0.073 |
|  |  |  |  |  |  |  |
| **AO** | **lM1-rM1** | 0.279, 0.094 | 0.302, 0.130 | 0.344, 0.096 | 0.337, 0.072 | 0.376, 0.112 |
|  | **lM1-SMA** | 0.166, 0.056 | 0.170, 0.071 | 0.384, 0.109 | 0.394, 0.121 | 0.411, 0.170 |
|  | **lM1-PMd** | 0.397, 0.078 | 0.416, 0.059 | 0.420, 0.084 | 0.448, 0.089 | 0.448, 0.151 |
|  | **lM1-Pr** | 0.335, 0.108 | 0.340, 0.126 | 0.423, 0.056 | 0.427, 0.062 | 0.462, 0.091 |

Values presented as mean, standard deviation. A, aerobic exercise priming; AO, action observation priming
