# Supplementary material for: Aerobic exercise and action observation priming modulate functional connectivity
Source: PLoS One. 2023 Apr 6;18(4):e0283975. doi: 10.1371/journal.pone.0283975 (PMC10079047; doi:10.1371/journal.pone.0283975)
Supplement: Attachment — Submitted filename: Reviewer_comments.docx [file pone.0283975.s005.docx]

Reviewer comments for the manuscript *Aerobic exercise and action observation priming modulate functional connectivity*

Interesting paper which opens a potential direction for rehabilitation: movement observation. I’ve been waiting for that branch to be better studied since the rehabilitative effects of sound observation are already relatively widely explored, like music listening in stroke and Parkinson rehabilitation. Therefore, the approach of this paper is relevant and timely.

I have a few remarks.

1. As the authors state, the sample size is very small. I’m concerned how much we can say anything about the EEG results due to the lack of statistical power. Since the participants were healthy adults (I’d prefer a more general term “healthy” rather than “unimpaired” in the abstract), which are relatively easy to recruit, why the researchers did not recruit more participants to this study?
2. If the aim is to use the gained results in rehabilitative purposes, why did the researchers include healthy walking by the participants themselves into the video-recorded walking observation? Other studies show that the perception of one’s own body evokes different brain processes than watching someone else’s body. To my knowledge, only schizophrenic patients are shown to lack such a separation when observing oneself and others’ bodies. It’s hard, if not impossible, to video record similar rhythmic and synchronized (“healthy”) walking from the patients (like Parkinson or stroke) for rehabilitation with movement observation to replicate the study setting of the current study in a rehabilitative context, which seems to be the motivation of this pilot study. The EEG results might change depending on whether the participants watch themselves or other people walking. As an attentional control, participants could have, for example, counted how many different people they saw walking, and leave out the videos of the participants themselves.
3. Interesting state in between movement observation and aerobic movement would be low intensity movement, especially when thinking about the trajectory in the rehabilitation process of the patients. Low and high intensity movements are shown to active different neural networks (e.g. Schmitt et al., 2019). Please, contemplate this “state-in-between” shortly in your paper.

Schmitt et al., 2019. Modulation of distinct intrinsic resting state brain networks by acute exercise bouts of differing intensity. Brain Plast.
