# Supplementary material for: Aerobic exercise and action observation priming modulate functional connectivity
Source: PLoS One. 2023 Apr 6;18(4):e0283975. doi: 10.1371/journal.pone.0283975 (PMC10079047; doi:10.1371/journal.pone.0283975)
Supplement: Attachment — Submitted filename: Response-to-Reviewers_2-15-23.docx [file pone.0283975.s006.docx]

**We appreciated the thoughtful comments from our two reviewers. My research team and I carefully implemented their feedback and made substantial revisions. Unfortunately, adding additional participants is not possible at this time. While this news may be understandably disappointing to the reviewers, I would like to emphasize our cross-over study design where each participant completed a series of EEG recordings that amounted to nearly 100 EEG recordings in our data set. Utilizing serial EEG recordings in this manner to elucidate the potential effects of two clinic-ready modes of priming on functional connectivity underscores the novelty of this work and certainly welcomes follow-up work involving larger cohorts and clinical populations.**

Comments from Editor:

## -not meet point 3 (3. Experiments, statistics, and other analyses are performed to a high technical standard and are described in sufficient detail.)

## We’ve made substantial edits to our manuscript to meet point 3. These changes include: [1] additional detail of our experimental parameters, [2] more references to substantiate our methodology, and [3] greater in-depth discussion of related work also featuring similar sample sizes. These revisions are explained in greater detail below.

## -more detail on the G-Power analysis conducted and the link to other studies as suggested by reviewer 2

## We decided that a formal power analysis was not appropriate at this time given the pilot nature of this work. However, per Reviewer 2’s recommendation, we describe other related EEG studies with similar sample sizes as ours whose experimental parameters and findings bolster the reliability of our work. Please refer to the last paragraph in the *Statistical Analysis* section.

## -provide the currently missing rationales for the technical and methodological choices made

## We added several additional references and rationale per Reviewer 2’s recommendation (please see our explanation to Reviewer 2 below).

**-**address the role of ‘priming’: Your study is on the effect of a motor related activity (aerobic exercise or action observation) on resting state functional connectivity, but you are setting it within the framework of 'priming'. The rationale for this needs to be clearer. Please explain in the manuscript why you are using the term 'priming' when you have not included a task that either preceded or followed the treatment

**We are not studying an intervention, but, rather, a pre-intervention. Throughout our Introduction section, we cite several promising articles that combine a bout of priming with a subsequent activity (usually a motor training paradigm) and observe enhanced learning (i.e. plasticity) with the addition of priming. This work is different in that we are interested in examining only the priming component and not the effects of priming + intervention. Understanding the underlying mechanisms associated with priming may optimize the outcomes arising from the pairing of priming + intervention. We added additional clarification in the Introduction section and adjusted our wording throughout the manuscript to not refer to aerobic- and action observation-based priming as “interventions” or “treatments.”**

-Similarly, the rationale for the choice of the two interventions is limited. Please provide a stronger rationale for including both conditions in the same study protocol.

**To reinforce our above response, we do not consider these to be interventions, but, rather priming conditions intended to be paired with physical training thereafter.** **We chose both aerobic exercise and action observation because of their potential for immediate implementation to a clinical environment. For some patients, aerobic exercise priming may not be possible or safe. Action observation may thus serve as a viable alternative. We emphasized this point in our Introduction section to better justify the need to study both in this study and “draw clearer lines” between the two priming modes. We did not intend to study the interaction or pairing of these priming modes.**

-Finally, you refer to the unbalanced order due to the randomization. Why have you not taken this into account as a factor in the model? This could have addressed this issue.

**Our initial manuscript mentioned controlling for the effects of priming order (i.e. sequence):**

***We assessed pre timepoints for significant treatment carryover and period effects by running the model with treatment, period, and sequence as fixed effects. We also screened for unequal treatment carryover, period effects, and treatment-by-period interactions by running the main model with treatment, period, and sequence as fixed effects.***

**We updated the statistical methods section and strengths/limitations section accordingly by adding the following statement.**

***We also recognize that six of the nine participants were randomized to the A-AO sequence; however, the non-significant effect of sequence when added to the model mitigated this issue.***

Reviewer 1: Interesting paper which opens a potential direction for rehabilitation: movement observation. I’ve been waiting for that branch to be better studied since the rehabilitative effects of sound observation are already relatively widely explored, like music listening in stroke and Parkinson rehabilitation. Therefore, the approach of this paper is relevant and timely.

1. As the authors state, the sample size is very small. I’m concerned how much we can say anything about the EEG results due to the lack of statistical power. Since the participants were healthy adults (I’d prefer a more general term “healthy” rather than “unimpaired” in the abstract), which are relatively easy to recruit, why the researchers did not recruit more participants to this study?

**Our research team contains several clinicians, and we collectively prefer “unimpaired” vs. “healthy” as it is frequently utilized in our respective medical fields.**

**We understand the concerns of the reviewer regarding sample size, and we agree that individuals without impairment are typically easier to recruit and enroll. However, we ask that the editor and the reviewers please consider the following points:**

- **Our cross-over study design involved the completion of 10 EEG recordings per participant which equates to nearly 100 EEG recordings in our data set. This necessitates considerable time and effort with data collection and analysis for each participant (approximately 8-10 hours/participant).**
- **This study occurred prior to Covid, and we are no longer conducting this study as several personnel involved in this work are no longer available. Enrolling additional participants is not possible at this time.**
- **Our statistical procedures are sound, and our language/writing throughout the manuscript when disseminating and interpreting our findings is appropriate and not inflated.**

2. If the aim is to use the gained results in rehabilitative purposes, why did the researchers include healthy walking by the participants themselves into the video-recorded walking observation? Other studies show that the perception of one’s own body evokes different brain processes than watching someone else’s body. To my knowledge, only schizophrenic patients are shown to lack such a separation when observing oneself and others’ bodies. It’s hard, if not impossible, to video record similar rhythmic and synchronized (“healthy”) walking from the patients (like Parkinson or stroke) for rehabilitation with movement observation to replicate the study setting of the current study in a rehabilitative context, which seems to be the motivation of this pilot study. The EEG results might change depending on whether the participants watch themselves or other people walking. As an attentional control, participants could have, for example, counted how many different people they saw walking, and leave out the videos of the participants themselves.

**We believe that there are several strategies for promoting consistent attentional state during the action observation-based priming condition. This, indeed, includes the reviewer’s suggestion of participants counting the total number of different individuals viewed.**

**As noted by the reviewer, our ultimate goal with this pilot work is implementation to a stroke rehabilitation setting. We chose the strategy that we did to align with future clinical work which would entail persons with stroke viewing clips of typical gait patterns (i.e. other individuals) and occasional clips of themselves (i.e. atypical gait patterns). Since the goal of action observation is to observe purposeful action to eventually mimic/replicate through practice, watching clips mostly of typical walking patterns with occasional clips of atypical walking patterns and asking individuals to acknowledge the number of atypical gait occurrences would be appropriate and clinically-relevant. The reviewer’s point of different brain processes involved in watching other’s vs. one’s own body is important, and we acknowledge it along with our above reasoning in the Discussion section (p. 23 in tracked-changes manuscript version) along with the addition of the Watanabe et al (2011) paper showing different brain activity evoked by different perspectives.**

- **Watanabe R, Watanabe S, Kuruma H, Murakami Y, Seno A, Matsuda T. Neural activation during imitation of movements presented from four different perspectives: a functional magnetic resonance imaging study. Neurosci Lett. 2011 Oct 3;503(2):100-4. doi: 10.1016/j.neulet.2011.08.016. Epub 2011 Aug 17. PMID: 21871533.**

**We also deleted the following sentence in our Methods section since participants, for the most part, viewed clips of others walking (75%, 15 out of 20 clips) during the action observation priming condition.**

***By targeting a relevant action (walking) and promoting selective attention (inclusion of participant-centered clips), we captured key action observation ingredients.***

3. Interesting state in between movement observation and aerobic movement would be low intensity movement, especially when thinking about the trajectory in the rehabilitation process of the patients. Low and high intensity movements are shown to active different neural networks (e.g. Schmitt et al., 2019). Please, contemplate this “state-in-between” shortly in your paper.

Schmitt et al., 2019. Modulation of distinct intrinsic resting state brain networks by acute exercise bouts of differing intensity. Brain Plast.

**We thank the reviewer for recognizing this paper. We agree with the reviewer that it merits discussion. We therefore cited and discussed the paper with regards to our findings (p. 21 of tracked-changes manuscript version).**

Reviewer 2: The authors presented a study investigating the priming effect of action observation and aerobic exercise on modulating functional connectivity between primary motor and motor-related areas during resting-state. They recorded EEG before and after (three times) priming and reported increased coherence in the alpha and beta frequency bands following both priming sessions. The manuscript is well-organized, the language is appropriate, and the topic is engaging. I appreciated the battery of tests used to evaluate the participants, the "strengths and limitations" section, and the goal of testing a short period of priming to be used in clinical settings. That said, I encourage the authors to consider a series of points that could strengthen their work that, in my opinion, is not ready for publication at this stage of work.

- The main point of concern regards the study's statistical power due to a quite limited number of participants and the 4x2 experimental design. The authors reported such an issue in the "Strengths and limitations" section, which I appreciated, but I'm not entirely convinced that acknowledging it solved the problem. The authors also reported a power analysis based on a previous study. However, such a study was conducted more than 25 years ago when the standards for reproducibility were different. Also, in that study, the authors recorded brain activity via a single channel and did not perform a coherence analysis between electrodes. I suggest the authors better structure this section of the manuscript, reporting, for instance, further examples of studies with similar sample sizes and discussing their results according to this point. The possibility of increasing the sample size would ideally represent a perfect solution. Given the future potential of using short priming sessions in clinical settings, the reliability of the results is essential.

**Consistent with our above response to Reviewer 1, unfortunately, increasing our sample size is not possible at this time. However, as recommended by this reviewer, we restructured this section of the manuscript by eliminating text related to the formal power analysis in G-plot given the pilot nature of this work where a formal power analysis is inappropriate. Instead, we now provide examples of several more recent EEG priming studies that have similar sample sizes (n=6 to 11) as ours. Though these papers feature a different EEG outcome measure to examine the effects of priming (event-related desynchronization) than ours, these papers collectively demonstrate significant changes in EEG activity following short-term periods of action observation priming utilizing EEG systems ranging from 19-64 leads. Importantly, these papers provide ample detail to honor standards for reproducibility which the initially cited paper lacked. The paper by Tani et al. is also noteworthy as it discusses obstacles of heterogeneity in clinical presentation within their small clinical cohort.**

- **Sun, Ziqi, et al. "Short-term Priming Effects: an EEG Study of Action Observation on Motor Imagery." IEEE Transactions on Cognitive and Developmental Systems (2022).**
- **Tani, Marina, et al. "Action observation facilitates motor cortical activity in patients with stroke and hemiplegia." Neuroscience Research 133 (2018): 7-14.**

- Please consider reporting more detail regarding the action observation priming (e.g., the size of the stimuli and distance from the screen).

**We added additional detail concerning action observation set-up and methodology to the revised manuscript.**

- Please consider reporting references in support of the choice of the "predefined electrodes" used for each region of interest (e.g., C3 for M1), and report the actual names/numbers of the electrodes considered (e.g., "surrounding six leads").

**We created a Supplementary table listing all predefined regions and corresponding electrode numbers and added relevant citations in the manuscript that further support this methodology.**

- **Cassidy JM, Wodeyar A, Wu J, Kaur K, Masuda AK, Srinivasan R, et al. Low-Frequency Oscillations Are a Biomarker of Injury and Recovery After Stroke. Stroke. 2020;51(5):1442-50.**
- **Cassidy JM, Wodeyar A, Srinivasan R, Cramer SC. Coherent neural oscillations inform early stroke motor recovery. Human Brain Mapping. 2021;42(17):5636-47.**
- **Homan RW, Herman J, Purdy P. Cerebral location of international 10-20 system electrode placement. Electroencephalogr Clin Neurophysiol. 1987;66(4):376-82.**
- **Zhou RJ, Hondori HM, Khademi M, Cassidy JM, Wu KM, Yang DZ, et al. Predicting Gains With Visuospatial Training After Stroke Using an EEG Measure of Frontoparietal Circuit Function. Front Neurol. 2018;9:597.**

- Please consider reporting references (and rationale) supporting the authors' choice throughout the study, for instance, for the use of the questionnaires/tests, frequency bands selected, and other technical/methodological decisions.

**Because future work involves the [1] implementation of aerobic- and action observation-based priming strategies to a clinical setting and [2] pairing these modes of priming with a physical training intervention, we included tests/measures that assess factors that may either influence one’s response to these priming strategies and/or subsequent motor learning when priming is paired with an intervention. We provided additional explanation in the manuscript accompanied by the following references:**

**Mood: Player MJ, Taylor JL, Weickert CS, Alonzo A, Sachdev P, Martin D, Mitchell PB, Loo CK. Neuroplasticity in depressed individuals compared with healthy controls. Neuropsychopharmacology. 2013 Oct;38(11):2101-8.**

**Trailmaking Test B: Tani M, Ono Y, Matsubara M, Ohmatsu S, Yukawa Y, Kohno M, et al. Action observation facilitates motor cortical activity in patients with stroke and hemiplegia. Neurosci Res. 2018;133:7-14.**

**(ABC Scale) Self-efficacy: Wulf G, Lewthwaite R. Optimizing performance through intrinsic motivation and attention for learning: The OPTIMAL theory of motor learning. Psychon Bull Rev. 2016;23(5):1382-414.**

**9HPT: Sainburg RL. Convergent models of handedness and brain lateralization. Front Psychol. 2014;5:1092.**

**We also added additional explanation and references justifying our choice in frequency bands and cortical regions.**

- Please consider reporting a graphic representation of the significant results (e.g., boxplots, raincloud plots) to help the reader understand the differences between conditions (e.g., pre, post, post10, etc.). It is not completely clear if the differences reported and discussed between post10, post20 and post30 were found only in the high beta band after exercise priming. A rewording of the "results" section to clarify postdoc interactions would be helpful.

**Initially, we chose to illustrate our findings (both significant and non-significant) using a series of topographs (Figure 2). This strategy enabled us to convey our results across multiple frequency bands, priming conditions, cortical regions, and timepoints. However, in line with the reviewer’s comment, we wanted to better illustrate individual data points along with group averages. We therefore created a series of bar graphs (Figures 3-6) to accompany Figure 2 and moved Tables 2-4 in the original manuscript to the Supplementary section.**

**We also reworded and reorganized the results section to improve upon the clarity of posthoc interactions and demonstrate consistency with our newly created figures.**
